# Postpartum Venous Thromboembolism: Altitudinal Gradients, Decadal Trends, and PE‐Specific Risk Profiling in Highland Populations

**DOI:** 10.1155/carj/3703610

**Published:** 2026-06-28

**Authors:** Jianbo Yu, Lobsang Chodron, Tenzin Chodron, Jo’nga Cering, Peiliang Gao, Xiaoxiao Zheng, Fang-e Shi, Zhenzhong Yang, Lobsang Cering, Guiying Dong

**Affiliations:** ^1^ Emergency Department, People’s Hospital of Xizang Autonomous Region, Lhasa, China; ^2^ Emergency Department, Peking University People’s Hospital, Beijing, China, pku.edu.cn

**Keywords:** highland, puerperium, pulmonary embolism, venous thromboembolism

## Abstract

**Background:**

This study primarily analyzed differences in venous thromboembolism (VTE) characteristics across altitudes and their temporal trends during the puerperium. Additionally, it identified independent risk factors for postpartum pulmonary embolism (PE).

**Methods:**

This retrospective study reviewed all postpartum VTE cases at People’s Hospital of Xizang Autonomous Region between 2015 and 2024.

**Results:**

This cohort study of 172 postpartum women (median age 31 [IQR 26–36]) compared high‐altitude (HA, *n* = 109) and very high‐altitude (VHA, *n* = 63) groups. PE proportion was significantly higher in VHA vs. HA *(* *p* = 0.033). VHA subjects also showed significant elevated preterm delivery (*p* = 0.033) and ≥ 3 deliveries (*p* < 0.001). Joinpoint regression (2015–2024) revealed biphasic trends: significant early‐phase escalations in VTE (APC = 18.05%) and deep vein thrombosis (DVT) (APC = 19.66%) during 2015–2022 (*p* < 0.05), followed by clinically relevant (though statistically nonsignificant) late‐phase reductions. PE proportion demonstrated a significant overall increase (APC = 18.77%, *p* < 0.05). In multivariate analysis, four independent predictors significantly increased PE risk: altitude gradient (OR 1.035, *p* < 0.001), multiparity (OR 2.548, *p* = 0.004), hypertension or eclampsia (OR 1.797, *p* = 0.001), and structural heart disease (OR 1.988, *p* < 0.001).

**Conclusion:**

This decade‐long analysis (2015–2024) revealed significant altitudinal gradients in postpartum VTE. Clinically significant escalation of postpartum VTE burden in high‐altitude populations warrants urgent intervention. Integrated multiparity management and enhanced comorbidity control are critical future initiatives for resolving key perinatal thrombotic risk bottlenecks.

## 1. Introduction

Venous thromboembolism (VTE), particularly its fatal pulmonary embolism (PE) subtype, represents a critical challenge in maternal health systems globally [1]. VTE persists as a predominant cause of maternal mortality during gestation and postpartum, while also conferring risks of permanent functional limitations [2]. Globally, PE accounts for 3% of all maternal deaths [3], with pregnancy‐associated VTE (PA–VTE) incidence ranging from 1/1000–3000 pregnancies [4, 5]. In developed nations, PE is the principal contributor to direct maternal mortality, exhibiting an incidence of 1.08/100,000 maternities [6] and causing 10%–15% of maternal deaths across Europe and North America [3, 5, 7–9]. Despite comparatively lower baseline rates of PA–VTE among Asian populations, contemporary epidemiological surveillance reveals a significant upward trajectory in incidence [10].

VTE risk progressively increases during gestation, peaking in the postpartum period [2]. A recent systematic review and meta‐analysis of studies with adjudicated VTE diagnoses reported pooled incidence rates of 118 per 100,000 person‐years during antepartum versus 424 per 100,000 person‐years postpartum [11]. The hypercoagulable state characteristic of gestation physiologically instantiates Virchow’s triad through three cardinal mechanisms: (1) upregulation of procoagulant factors, (2) impaired fibrinolytic capacity, and (3) venous stasis induced by uterine compression [12]. This triad synergistically elevates thrombotic susceptibility, rendering pregnancy a 6‐fold higher VTE risk state compared to nonpregnant counterparts [13].

Current evidence confirms a substantially elevated risk of thromboembolic events at high and extreme altitudes [14, 15]. This phenomenon is exemplified by Indian military personnel stationed at 3600 m demonstrating a 24.5‐fold higher risk of DVT relative to low‐altitude counterparts [16]. USA high‐altitude (HA) cohorts demonstrated a 2‐fold elevation in thrombotic event incidence compared to sea‐level counterparts [17]. Domestic cohort studies reveal a significant elevation in PE incidence among HA residents compared to low‐altitude populations [18]. Hypoxia is recognized as a key trigger for prethrombotic alterations in coagulation function [19]. This prothrombotic state emerges through three primary mechanisms: (1) platelet proteomic modulation [20, 21], (2) enhanced clot formation and (3) fibrinolytic suppression [22]. Additionally, elevated erythrocyte counts and hemoglobin concentrations, recognized as independent thrombotic risk factors, contribute to this risk by (1) inducing endothelial damage, (2) increasing blood viscosity, and (3) elevating vascular resistance [23].

VTE incidence varies across population subgroups and risk profiles; moreover, racial and ethnic disparities in PA–VTE are well‐documented [12]. This elevated baseline PA–VTE risk is further compounded by additional determinants. Despite established knowledge on PA–PE risks, few studies have investigated temporal trends, regional variations, and postpartum PE risk in hypoxic settings. Identifying and evaluating potential risk factors is essential for accurate risk stratification and the formulation of effective preventive strategies. However, research specifically addressing PE risk in the HA postpartum population remains lacking in China. We hypothesize that both altitude and obstetric complications serve as significant independent predictors of postpartum PE. To address this gap, leveraging a decade of hospital admission data, this study analyzes epidemiological trajectories and associated risk factors for postpartum VTE in the Lhasa cohort.

## 2. Materials and Methods

### 2.1. Data Source and Study Population

This retrospective cohort study reviewed all consecutive emergency admissions at a 500‐bed university hospital (Jan 2015‐Dec 2024), serving as the regional apex referral center. Postpartum VTE cases were ascertained using ICD‐10‐I26.900 × 014 (acute PE), I80.303 or I80.207 (DVT), I26.900 × 001 (PE), O88.201 (obstetrical PE), and I26.900 × 015 (acute pulmonary thromboembolism). Due to the fact that DVT or PE is a deterministic diagnosis based on examination, each case was reviewed to assess the diagnostic certainty. If the patient meets the clear criteria for DVT/PE diagnosis, they will be included in the queue for full analysis. Cases were excluded due to a lack of clinical or imaging evidence to support the diagnosis.

The selection criteria were as follows: (a) age ≥ 18 years, (b) acute VTE per AHA 2018 criteria (symptomatic/incidental DVT by complete‐compression ultrasonography; PE by CTPA or high‐probability V/Q scan) [24], and (c) diagnosis ≤ 42 days postpartum. The exclusion criteria included (a) prior VTE history, (b) active thrombophilia (cancer, SLE, APS, or genetic thrombophilia), (c) rehospitalizations for a principal diagnosis identical to the initial hospitalization, and (d) unverifiable index event data.

**FIGURE 1:**
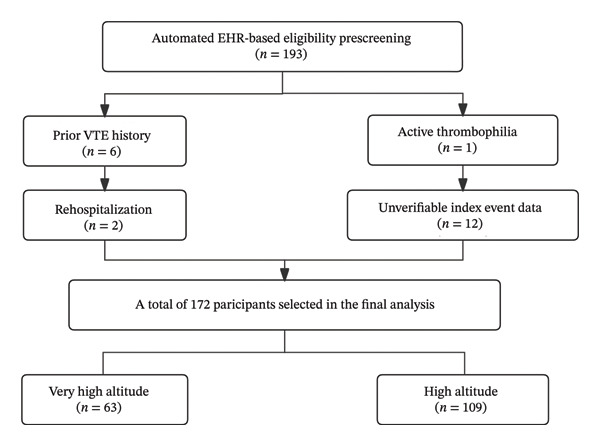
Participant flow through the study: Enrollment, allocation, and analysis cohorts (*N* = 172 screened).

Regions exceeding 2500 m above sea level elicit distinct physiological adaptations and are thus defined as plateaus [25]. Substantial discrepancies persist in altitude classification criteria across studies. According to Chinese academic standards, plateaus are stratified into: HA (2500–4500 m), super‐high altitude (4500–5500 m), and extremely high altitude (> 5500 m) [26]. Patient altitude exposure was determined during hospitalization by geocoding residential addresses. Within our cohort, the majority (≥ 95%) resided at 3500–4500 m, with minimal representation beyond this range. Based on this distribution, we adopted the 4000 m threshold [27] to stratify postpartum VTE cases into two groups: HA and very HA (VHA). Figure 1 delineates the patient screening workflow, encompassing eligibility assessment, exclusion rationale, and final cohort selection.

### 2.2. Variables

Baseline characteristics included demographics (age, ethnicity, geocoded altitude, occupation, and health insurance), body mass index (BMI), maternal comorbidities (e.g., pregnancy hypertension or eclampsia, structural heart disease, diabetes mellitus, moderate to severe anemia, and varicose veins), obstetric profile (e.g., delivery mode, peripartum events, and obstetric history), as well as fetal outcomes (preterm delivery, multiple pregnancy, and stillbirth), and serial laboratory monitoring was recorded. All data underwent dual independent verification by two physicians; discrepancies in interpretation were adjudicated by a third researcher.

### 2.3. Statistical Analysis

Statistical analyses were performed using IBM SPSS Statistics 26 Software. Normality of data distribution was assessed using the Kolmogorov–Smirnov test. Continuous variables are expressed as median (interquartile range [IQR]) and compared using the Mann–Whitney *U* test. Categorical variables are presented as *n* (%) and compared using the Pearson chi‐square test or Fisher’s exact test, as appropriate. Univariable and multivariable logistic regression analysis was employed to identify risk factors for PE. Variable selection was guided by prior evidence and clinical relevance. Multivariate analysis initially incorporated all univariate predictors significant at *p* < 0.05, followed by backward elimination to retain only independently significant predictors (final *p* < 0.05). Statistical significance was defined as two‐sided *p* values < 0.05.

Utilizing Joinpoint regression software (v5.1.0; National Cancer Institute, Bethesda, MD), we analyzed decade‐long trends in VTE/PE/DVT cases. Case counts underwent logarithmic transformation to construct a segmented continuous log‐linear model. Optimal fitting identified trend inflection points (maximum 5 joinpoints permitted, with ≥ 4 data points per segment ensuring model stability). Significance testing employed the Monte Carlo permutation method (4499 iterations, *α* = 0.05), calculating annual percent change (APC) with 95% confidence intervals (95% CI). Outputs included visualized trend trajectories and statistical tables detailing AAPC, *p*‐values, and associated metrics.

## 3. Results

### 3.1. Baseline Characteristics

This study included 30,652 pregnancies. A total of 193 cases of VTE were diagnosed, yielding an overall incidence of 5.61 per 1000 pregnancies; the VHA group accounted for 36.7% of these cases. Rigorous exclusion criteria were then applied to the 193 consecutively screened postpartum VTE cases: prior VTE history (*n* = 6; 3.5%), active thrombophilia confirmed by previous history (*n* = 1; 0.6%), hospital readmissions with identical principal diagnoses (*n* = 2; 1.2%), incomplete baseline phenotyping (*n* = 12; 7.0%). The analytic cohort comprised 172 women (median age 31 [IQR 26–36]), among whom 50 (29.1%) met criteria for advanced maternal age (≥ 35 years), with the following clinical profile: PE prevalence was 43 cases (25.0%), and 63 individuals (36.6%) resided in VHA areas. No mortality cases were documented in the study cohort.

### 3.2. Altitude‐Stratified Clinical Characteristics

Significant altitude gradient effects emerged in Table 1. Overall, VHA residents exhibited higher burdens of PE (34.9% vs. 19.3%; *p* = 0.033), preterm delivery (20.6% vs. 9.2%; *p* = 0.033), and multiparity (≥ 3 deliveries, 39.7% vs. 15.6%; *p* < 0.001).

**TABLE 1:** Elevation‐specific VTE clinical characteristics architecture.

	All (*n* = 172)	Very high altitude (*n* = 63)	High altitude (*n* = 109)	*p* value
Age, median (IQR), years	31 (26, 36)	32 (26, 36)	31 (28, 34)	0.790
Tibetan, *n* (%)	169 (98.3%)	63 (100%)	106 (97.2%)	0.300^F^
Non‐O blood group, *n* (%)	123 (71.5%)	43 (68.3%)	80 (73.4%)	0.472
BMI, median (IQR), kg/m^2^	26.3 (23.9, 29.3)	25.2 (23.9, 29.3)	26.6 (23.7, 30.0)	0.233
Smoker, *n* (%)	3 (1.7%)	1 (1.6%)	2 (1.8%)	0.261
Cesarean section, *n* (%)	123 (71.5%)	46 (73.0%)	77 (70.6%)	0.740
Multiparity, *n* (%)	123 (71.5%)	46 (73.0%)	80 (73.4%)	0.957
Advanced maternal age (≥ 35 years), *n* (%)	50 (29.1%)	18 (28.6%)	32 (29.4%)	0.913
Postpartum days, median (IQR), days	5 (3, 7)	5 (3, 6)	5 (3, 6)	0.831
Previous births ≥ 3, *n* (%)	42 (24.4%)	17 (39.7%)	25 (15.6%)	< 0.001^∗^
Pulmonary embolism, *n* (%)	43 (25.0%)	22 (34.9%)	21 (19.3%)	0.033^∗^

*Occupational Status*				
Secure pay, *n* (%)	42 (24.4%)	13 (20.6%)	29 (26.6%)	0.584
Agriculture/animal husbandry, *n* (%)	98 (57.0%)	39 (61.9%)	59 (54.1%)	
Unemployment, *n* (%)	32 (18.6%)	11 (17.5%)	21 (19.3%)	

*Health Insurance*				
National medical insurance, *n* (%)	42 (24.4%)	18 (20.6%)	29 (26.6%)	0.126
Rural cooperative medical care, *n* (%)	98 (57.0%)	3 (61.9%)	59 (54.1%)	
Self‐pay, *n* (%)	32 (18.6%)	42 (17.5%)	21 (19.3%)	

*Medical History*				
Pregnancy hypertension or Eclampsia, *n* (%)	26 (15.1%)	10 (15.9%)	16 (14.7%)	0.833
Diabetes mellitus, *n* (%)	3 (1.7%)	0 (0.0%)	3 (2.8%)	0.300^F^
Structural heart disease, *n* (%)	8 (4.7%)	4 (6.3%)	4 (3.7%)	0.466^F^
Moderate to severe anemia, *n* (%)	13 (7.6%)	7 (11.1%)	6 (5.5%)	0.180
Varicose veins, *n* (%)	15 (8.7%)	5 (7.9%)	10 (9.2%)	0.782

*Fetal Outcomes*				
Preterm delivery, *n* (%)	23 (13.4%)	13 (20.6%)	10 (9.2%)	0.033^∗^
Multiple pregnancy, *n* (%)	9 (5.2%)	4 (6.3%)	5 (4.6%)	0.726^F^
Stillbirth, *n* (%)	17 (9.9%)	9 (14.3%)	8 (7.3%)	0.141

*Peripartum Events*				
Transfusion packed cells volume, median (IQR), ml	200 (200, 250)	200 (200, 250)	200 (150, 250)	0.446
Pregnancy‐related infection, *n* (%)	39 (22.7%)	16 (25.4%)	23 (21.1%)	0.517

*Laboratory Examination*				
Hb (Altitude correction), median (IQR), g/L	96 (81, 113)	101 (77, 116)	96 (82, 109)	0.650
FIB, median (IQR), g/L	6.26 (3.86, 13.09)	6.20 (3.59, 12.87)	6.39 (3.86, 13.11)	0.769
D‐Dimer, median (IQR), mg/L	2.25 (1.36, 3.84)	1.77 (1.25, 4.05)	2.38 (1.40, 3.72)	0.534

*Note:* Data are presented as the median (IQR) or *n* (%). F, Fisher’s exact test.

^∗^ *p* < 0.05 was considered statistically significant.

### 3.3. Decadal Trend in Yearly Case Burden and Incidence

**FIGURE 2:**
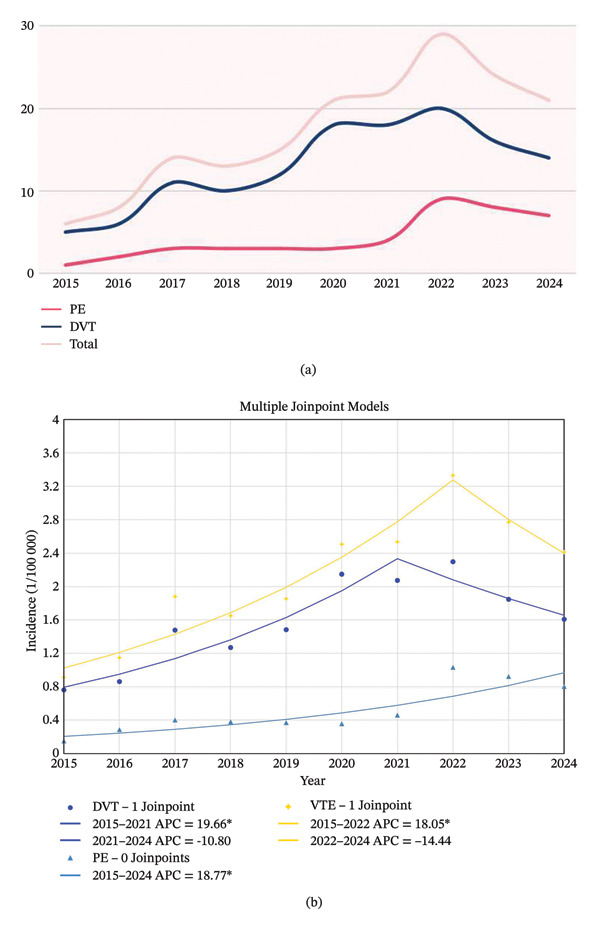
Decadal dynamics of annual disease burden and incidence rates: A population‐based temporal analysis (2015–2024).

Figure 2a illustrates the thromboembolic disease burden (2015–2024), showing a rise in VTE, DVT, and PE cases from initial lows (2015: 6, 5, 1) to peak cases (2022: 29, 20, 9). This represents an overall increase of 383% (VTE), 300% (DVT), and 800% (PE), with a mean near‐tripling of cases over 8 years. A subsequent reversal manifested post‐2022, with 2024 preliminary data indicating a 27.6% reduction in composite VTE events.

Joinpoint regression analysis (Figure 2b) delineated two distinct epidemiological phases. During the early accumulation phase (2015–2022 for VTE; 2015–2021 for DVT), both conditions showed significant annual increases: VTE rose at 18.05% per year (95% CI: 9.88–26.83; *p* < 0.05), while DVT increased at 19.66% annually (95% CI: 9.63–30.60; *p* < 0.05). Conversely, the late attenuation phase (2022–2024 for VTE; 2021–2024 for DVT) featured nonsignificant declines: VTE decreased at an annual rate of −14.44% (95% CI: −49.98 to 46.36; *p* > 0.05), and DVT fell at −10.80% per year (95% CI: −31.13 to 15.55; *p* > 0.05). PE incidence demonstrated a significant overall increase (APC = 18.77%; 95% CI: 10.75 to 27.37; *p* < 0.05).

### 3.4. Risk Factors for PE

Univariable logistic regression (Table 2) identified significant PE predictors in HA parturients: age (OR 1.477 [95% CI, 1.313–1.729], *p* = 0.001), altitude gradient (VHA vs HA, OR 1.108 [95% CI, 1.050–1.169], *p* < 0.001), multiparity (OR 2.767 [95% CI, 1.419–5.396], *p* = 0.003), cesarean section (OR 3.724 [95% CI, 1.182–11.731], *p* = 0.025), pregnancy‐related infection (OR 2.285 [95% CI, 1.262–4.136], *p* = 0.006), hypertension or eclampsia (OR 2.629 [95% CI, 1.558–4.437], *p* < 0.001), structural heart disease (OR 5.417 [95% CI, 2.303–12.738], *p* < 0.001), and D‐dimer (OR 1.009 [95% CI, 1.001–1.018], *p* = 0.028).

**TABLE 2:** Risk factors associated with PE.

Variables	Univariable OR (95% CI)	*p* value	Multivariable OR (95% CI)	*p* value
Age	1.477 (1.313, 1.729)	0.001		
Altitude gradient	1.108 (1.050, 1.169)	< 0.001	1.035 (0.995, 1.077)	< 0.001
BMI	1.039 (0.981, 1.100)	0.195		
Previous births	1.174 (0.746, 1.848)	0.488		
Multiparity	2.767 (1.419, 5.396)	0.003	2.548 (1.341, 4.840)	0.004
Cesarean section	3.724 (1.182, 11.731)	0.025		
Preterm delivery	1.113 (0.598, 2.072)	0.735		
Stillbirth	1.592 (0.748, 3.389)	0.227		
Multiple pregnancy	1.000 (1.000, 1.000)	0.490		
Transfusion volume	1.000 (0.999, 1.002)	0.706		
Pregnancy‐related infection	2.285 (1.262, 4.136)	0.006		
Non‐O blood group	0.992 (0.959, 1.025)	0.617		
Varicose veins	1.103 (1.031, 1.179)	0.004		
Hypertension or eclampsia	2.629 (1.558, 4.437)	< 0.001	1.797 (0.934, 3.457)	0.001
Diabetes mellitus	2.218 (0.489, 10.056)	0.302		
Structural heart disease	5.417 (2.303, 12.738)	< 0.001	1.988 (1.945, 2.032)	< 0.001
Moderate to severe anemia	0.661 (0.398, 1.095)	0.108		
Fibrinogen	1.002 (0.991, 1.013)	0.714		
D‐Dimer	1.009 (1.001, 1.018)	0.028		

*Note:* *p* < 0.05 was considered statistically significant.

In the multivariable logistic regression model altitude gradient (OR 1.035 [95% CI, 0.995–1.077], *p* < 0.001), multiparity (OR 2.548 [95% CI, 1.341–4.840], *p* = 0.004), hypertension or eclampsia (OR 1.797 [95% CI, 0.934–3.457], *p* = 0.001) and structural heart disease (OR 1.988 [95% CI, 1.945–2.032], *p* < 0.001) were associated with increased odds of PE.

## 4. Discussion

The diagnosis and management of postpartum VTE are confronting a dearth of high‐quality evidence‐based guidance [28]. This study partially addresses a critical evidence gap by delineating the altitude‐dependent risk gradient in postpartum VTE. Beyond the inherent postpartum hypercoagulability captured by Virchow’s triad [29], our findings corroborate the significantly amplified thromboembolic hazard in HA environments. In synthesis, HA‐induced thromboembolic disorders are unlikely to be attributable to a singular etiology. Consequently, this phenomenon should be conceptualized as a multifactorial phenotype. Further investigation is warranted to elucidate both the thrombotic risk dynamics at high altitude and the potential pathophysiological mechanisms involved [17].

In our cohort study from 2015 to 2024, there was no significant downward trend in the incidence of postpartum VTE/DVT, while postpartum PE showed an overall upward trend. Consistent with national reports, the overall incidence of VTE in China has shown an annual upward trend. While this may be partially attributed to heightened diagnostic awareness and the widespread adoption of imaging technologies, it is more likely a consequence of inadequate preventive measures. At the national level, a comprehensive meta‐analysis encompassing 53 studies and involving over 3.81 million pregnant women revealed a VTE incidence rate of 0.13% across China [30]. A series of complementary investigations have further revealed that the scale and resources of hospitals significantly influence the assessment of VTE risk and the incidence rate among pregnant women and those in the postpartum period [31–35]. Relative to low‐altitude areas, the escalating disease burden and incidence of postpartum VTE observed in HA, low‐population‐density regions were more deeply concerning. Notably, at the backdrop of China’s ongoing promotion of a comprehensive VTE care strategy centered on “heightening awareness, standardizing diagnosis and treatment, advancing in‐depth research, establishing robust systems, and strengthening management oversight”, the previously upward trajectory of incidence has shown signs of plateauing [36]. PE incidence in the general population demonstrated sustained decline, while the puerperium did not show the same positive trend, underscoring a critical need for far greater vigilance. The topic of “VTE risk assessment in pregnancy” at the ISTH conference held in Melbourne, Australia, in 2019 sounded the alarm for this group again [2].

In our study, patients in both the HA and VHA groups presented with gestational anemia [37] and demonstrated higher rates of preterm birth, cesarean section, and stillbirth. These findings align with existing literature. A meta‐analysis demonstrated that low maternal Hb (< 110 g/L) increased the odds of small‐for‐gestational‐age (SGA), stillbirth, preeclampsia, and gestational diabetes [38]. Similarly, another study indicated that both high and low hemoglobin levels were associated with adverse outcomes, specifically stillbirth, preterm birth, and SGA, identifying 11–13 g/dL as the optimal range [39]. Furthermore, anemia has been linked to a trend of increased cesarean delivery risk [40]. Notably, regarding the altitude factor, residing at high altitude was found to exert an independent adverse effect on pregnancy outcome [39]. In the specific context of China’s plateau regions, the prevalence of anemia is markedly high due to harsh environments, underdeveloped health infrastructure, and unique dietary habits (e.g., strong tea consumption). Research in these regions further confirms the association between gestational anemia and increased incidences of hypertensive disorders, cesarean section, and postpartum hemorrhage [41].

Investigating risk factors for postpartum PE specific to HA settings is critical for mitigating morbidity and mortality. The cesarean section, high altitude, gestational hypertension, and eclampsia, which were mentioned in the preceding paragraph, were confirmed as risk factors for PE. This study quantified the dose‐response relationship between altitude gradient and postpartum PE. Multiparity was identified as the strongest independent predictor. Future initiatives must address critical bottlenecks in perinatal thrombotic risk, including: (1) integrated management of multiparity and cesarean‐related cumulative trauma; (2) enhanced screening and control for comorbidities; and (3) optimization of tailored mechanical/pharmacologic prophylaxis protocols. These advancements are essential for implementing the HA maternal safety strategy.

Notwithstanding, this study has several noteworthy limitations. The niche focus on postpartum VTE and single‐center design resulted in a limited number of VTE events despite comprehensive 10‐year case ascertainment. Additionally, the retrospective nature inherently constrains causal inference due to unmeasured confounding. Crucially, findings are specific to HA postpartum populations, with a predominantly Tibetan cohort, limiting generalizability to other settings [42]. Moreover, the unavailability of control data from low‐altitude regions resulted in a lack of direct comparative datasets between high‐ and low‐altitude populations, limiting our ability to comprehensively elucidate the potential impact of altitude on postpartum PE risk. This limitation is also evident in the Hb levels of the study population; specifically, the significant impact of high altitude on blood indicators could not be demonstrated in risk factors. Multicenter collaborative studies are needed to further clarify this relationship and develop targeted prevention and treatment strategies for different populations. To address these gaps, future large‐scale prospective multicenter studies are imperative, particularly to establish evidence‐based VTE risk stratification models for Chinese perinatal women [43]. Such efforts will enable developing standardized clinical guidelines and integrated management systems for VTE screening and prevention across diverse healthcare tiers.

## 5. Conclusions

This decade‐long analysis reveals significant altitude‐gradient disparities in postpartum VTE profiles, sustained high VTE/DVT incidence versus accelerating PE trends, driven by specific risk integrations, such as maternal parturition status, obstetric complications, and also HA exposure.

## Author Contributions

Conceptualization, methodology: Guiying Dong; writing: Jianbo Yu; formal analysis: Fang‐e Shi and Xiaoxiao Zheng; resources: Jo’nga Cering, Lobsang Chodron, Tenzin Chodron, and Peiliang Gao; review and editing: Lobsang Cering and Zhenzhong Yang; funding: Guiying Dong.

## Funding

This work was supported by the grant XZZR202402133(W) from the Xizang Natural Science Foundation, Group Medical Aid to Xizang Medical Proiect Funding.

## Disclosure

All authors have read and agreed to the published version of the manuscript.

## Ethics Statement

This study was approved by the Ethics Committee of People’s Hospital of Xizang Autonomous Region (Approval No. ME‐TBHP‐24–055) and the study was performed in accordance with the ethical standards as laid down in the 1964 Declaration of Helsinki and its later amendments or comparable ethical standards. Given the retrospective nature of the study, informed consent was exempted.

## Consent

The authors have nothing to report.

## Conflicts of Interest

The authors declare no conflicts of interest.

## Data Availability

The datasets and resources analyzed during the current study are available from the corresponding authors upon reasonable request.

## References

[1] Farmakis I. T. , Barco S. , Hobohm L. et al., Maternal Mortality Related to Pulmonary Embolism in the United States, 2003–2020, American Journal of Obstetrics & Gynecology MFM. (2023) 5, no. 1, 10.1016/j.ajogmf.2022.100754. 36155111

[2] Ewins K. and Ni Ainle F. , VTE Risk Assessment in Pregnancy, Research and Practice in Thrombosis and Haemostasis. (2020) 4, no. 2, 183–192, 10.1002/rth2.12290. 32110748 PMC7040539

[3] Dado C. D. , Levinson A. T. , and Bourjeily G. , Pregnancy and Pulmonary Embolism, Clinics in Chest Medicine. (2018) 39, no. 3, 525–537, 10.1016/j.ccm.2018.04.007. 30122177 PMC8018832

[4] Wiegers H. M. G. and Middeldorp S. , Contemporary Best Practice in the Management of Pulmonary Embolism During Pregnancy, Therapeutic Advances in Respiratory Disease. (2020) 14, 10.1177/1753466620914222. PMC7238314 32425105

[5] Blondon M. , Martinez de Tejada B. , Glauser F. , Righini M. , and Robert-Ebadi H. , Management of High-Risk Pulmonary Embolism in Pregnancy, Thrombosis Research. (2021) 204, 57–65, 10.1016/j.thromres.2021.05.019. 34146979

[6] Simcox L. E. , Ormesher L. , Tower C. , and Greer I. A. , Pulmonary thrombo-embolism in Pregnancy: Diagnosis and Management, Breathe. (2015) 11, no. 4, 282–289, 10.1183/20734735.008815. 27066121 PMC4818214

[7] Knight M. , Ukoss. Antenatal Pulmonary Embolism: Risk Factors, Management and Outcomes, BJOG. (2008) 115, no. 4, 453–461, 10.1111/j.1471-0528.2007.01622.x. 18201281

[8] Magee L. A. , Arya R. , Boag C. et al., Pregnancy-Related Venous Thromboembolism Risk Perception and Prevention in Risk-Averse Times-Significant Change Required: A Commentary, BJOG. (2025) 132, no. 10, 1341–1345, 10.1111/1471-0528.18229. 40400112 PMC12315063

[9] Bukhari S. , Fatima S. , Barakat A. F. , Fogerty A. E. , Weinberg I. , and Elgendy I. Y. , Venous Thromboembolism During Pregnancy and Postpartum Period, European Journal of Internal Medicine. (2022) 97, 8–17, 10.1016/j.ejim.2021.12.013. 34949492

[10] Tsai C. T. and Chao T. F. , Incidence and Risk Factors for Pregnancy-Associated Venous Thromboembolism: Are There Differences Between East and West?, Thrombosis and Haemostasis. (2023) 123, no. 09, 911–912, 10.1055/s-0043-1769736. 37276880

[11] Abdul Sultan A. , Tata L. J. , Grainge M. J. , and West J. , The Incidence of First Venous Thromboembolism in and Around Pregnancy Using Linked Primary and Secondary Care Data: a Population Based Cohort Study from England and Comparative meta-analysis, PLoS One. (2013) 8, no. 7, 10.1371/journal.pone.0070310. PMC3726432 23922975

[12] Varrias D. , Spanos M. , Kokkinidis D. G. , Zoumpourlis P. , and Kalaitzopoulos D. R. , Venous Thromboembolism in Pregnancy: Challenges and Solutions, Vascular Health and Risk Management. (2023) 19, 469–484, 10.2147/VHRM.S404537. 37492280 PMC10364824

[13] Parunov L. A. , Soshitova N. P. , Ovanesov M. V. , Panteleev M. A. , and Serebriyskiy I. I. , Epidemiology of Venous Thromboembolism (VTE) Associated With Pregnancy, Birth Defects Research Part C: Embryo Today. (2015) 105, no. 3, 167–184, 10.1002/bdrc.21105. 26406886

[14] Dai L. , Zuo Q. , Chen F. , Chen L. , and Shen Y. , The Association and Influencing Factors Between Antipsychotics Exposure and the Risk of VTE and PE: a Systematic Review and Meta-analysis, Current Drug Targets. (2020) 21, no. 9, 930–942, 10.2174/1389450121666200422084414. 32321400

[15] Sha Y. , Zhang J. , Ci Y. et al., Cerebral Venous Thrombosis at High Altitude: More Severe Symptoms and Specific Predisposing Factors Than Plain Areas, Thrombosis Journal. (2024) 22, no. 1, 10.1186/s12959-024-00643-2. PMC11308695 39118154

[16] Trunk A. D. , Rondina M. T. , and Kaplan D. A. , Venous Thromboembolism at High Altitude: Our Approach to Patients at Risk, High Altitude Medicine & Biology. (2019) 20, no. 4, 331–336, 10.1089/ham.2019.0049. 31479310

[17] Gupta N. and Ashraf M. Z. , Exposure to High Altitude: a Risk Factor for Venous Thromboembolism?, Seminars in Thrombosis and Hemostasis. (2012) 38, no. 02, 156–163, 10.1055/s-0032-1301413. 22422330

[18] Wu J. , Zhang J. , Wang R. et al., Clinical Characteristics of Pulmonary Embolism at Extremely High Altitude: A Single-Center Retrospective Study, Frontiers in Public Health. (2025) 13, 10.3389/fpubh.2025.1453700. PMC11983428 40213421

[19] Kammerer T. , Walzl A. , Müller T. et al., Effects of Hypobaric Hypoxia on Coagulation in Healthy Subjects Exposed to 3500 M Altitude, High Altitude Medicine & Biology. (2023) 24, no. 2, 94–103, 10.1089/ham.2022.0154. 37339401

[20] Jha P. K. , Sahu A. , Prabhakar A. et al., Genome-Wide Expression Analysis Suggests Hypoxia-Triggered Hyper-Coagulation Leading to Venous Thrombosis at High Altitude, Thrombosis and Haemostasis. (2018) 118, no. 07, 1279–1295, 10.1055/s-0038-1657770. 29864786

[21] Rocke A. S. , Paterson G. , Barber M. et al., Thromboelastometry and Platelet Function During Acclimatization to High Altitude, Thrombosis and Haemostasis. (2018) 118, no. 01, 63–71, 10.1160/TH17-02-0138. 29304526 PMC6260116

[22] Jiang P. , Wang Z. , Yu X. et al., Effects of Long-Term High-Altitude Exposure on Fibrinolytic System, Hematology. (2021) 26, no. 1, 503–509, 10.1080/16078454.2021.1946265. 34238131

[23] Tremblay J. C. , Hoiland R. L. , Howe C. A. et al., Global REACH 2018: High Blood Viscosity and Hemoglobin Concentration Contribute to Reduced Flow-Mediated Dilation in High-Altitude Excessive Erythrocytosis, Hypertension. (2019) 73, no. 6, 1327–1335, 10.1161/HYPERTENSIONAHA.119.12780. 31006327

[24] Bates S. M. , Rajasekhar A. , Middeldorp S. et al., American Society of Hematology 2018 Guidelines for Management of Venous Thromboembolism: Venous Thromboembolism in the Context of Pregnancy, Blood Advances. (2018) 2, no. 22, 3317–3359, 10.1182/bloodadvances.2018024802. 30482767 PMC6258928

[25] Luks A. M. , Beidleman B. A. , Freer L. et al., Wilderness Medical Society Clinical Practice Guidelines for the Prevention, Diagnosis, and Treatment of Acute Altitude Illness: 2024 Update, Wilderness and Environmental Medicine. (2024) 35, no. 1, 2S–19S, 10.1016/j.wem.2023.05.013. 37833187

[26] Committee E , Expert Consensus on Clinical Application of Blood Glucose Monitoring in High-Altitude Areas of China (2024 Edition), Chinese Journal of diabetes. (2024) 16, 1207–1214, 10.3760/cma.j.cn115791-20240702-00348.

[27] Implementation Measures for Subsidies for Difficult and Remote Areas of Government Agencies and Institutions , Bulletin of the State Council of the People’s Republic of China, 1993, 1538–1539.

[28] Ephraums S. , Dasgupta A. , Korah S. , Pasupathy D. , and Seeho S. , A Comparison of International Clinical Practice Guidelines for Postpartum Venous Thromboembolism Prophylaxis, BMC Pregnancy and Childbirth. (2025) 25, no. 1, 10.1186/s12884-025-07246-3. PMC11823154 39939968

[29] Frank A. K. and Samuelson Bannow B. , Venous Thromboembolism in Pregnancy and Postpartum: An Illustrated Review, Research and Practice in Thrombosis and Haemostasis. (2024) 8, no. 4, 10.1016/j.rpth.2024.102446. PMC11263788 39045339

[30] Luo X. , Shan D. , Zhang L. et al., Incidence of Maternal Venous Thromboembolism in China: a Systematic Review and Meta-Analysis, International Journal of Gynaecology & Obstetrics. (2023) 163, no. 1, 75–88, 10.1002/ijgo.14776. 37069776

[31] Ge Y. Z. , Zhang C. , Cai Y. Q. , and Huang H. F. , Application of the RCOG Risk Assessment Model for Evaluating Postpartum Venous Thromboembolism in Chinese Women: A Case-Control Study, Medical Science Monitor. (2021) 27, 10.12659/MSM.929904. PMC8274362 34230447

[32] Zhou Q. , Zhao Z. , Xu J. , Xiong Y. , and Li X. , Hospital Variation and Associated Organizational Factors of Pregnancy-Related Venous Thromboembolism in China, Clinical and Applied Thrombosis/Hemostasis. (2022) 28, 10.1177/10760296221076148. PMC8980411 35360953

[33] Zhao Z. , Zhou Q. , and Li X. , Missed Opportunities for Venous Thromboembolism Prophylaxis During Pregnancy and the Postpartum Period: Evidence from Mainland China in 2019, BMC Pregnancy and Childbirth. (2021) 21, no. 1, 10.1186/s12884-021-03863-w. PMC8142288 34030656

[34] Liu S. , Tang F. , Zhong X. et al., Knowledge, Attitudes, and Practices on Venous Thromboembolism Among Maternal Women and Analysis of Influencing Factors: a cross-sectional Study in Southwest China, BMC Pregnancy and Childbirth. (2025) 26, no. 1, 10.1186/s12884-025-08570-4. PMC12821795 41402762

[35] Wang Z. L. et al., [Survey of Related Factors of Maternal Venous Thromboembolism in Nine Hospitals of China], Zhonghua Fu Chan Ke Za Zhi. (2020) 55, 667–672, 10.3760/cma.j.cn112141-20200414-00326. 33120477

[36] Zhai Z. G. , Jia X. D. , Ma C. , and Wang C. , Consolidate the Foundation, Steadily Promote and Comprehensively Improve the Quality Control in Prevention and Management of Venous Thromboembolism in China, Zhonghua Yixue Zazhi. (2022) 102, 3333–3337, 10.3760/cma.j.cn112137-20220903-01866. 36372766

[37] McLean E. , Cogswell M. , Egli I. , Wojdyla D. , and de Benoist B. , Worldwide Prevalence of Anaemia, WHO Vitamin and Mineral Nutrition Information System, 1993-2005, Public Health Nutrition. (2009) 12, no. 4, 444–454, 10.1017/S1368980008002401. 18498676

[38] Young M. F. , Oaks B. M. , Tandon S. , Martorell R. , Dewey K. G. , and Wendt A. S. , Maternal Hemoglobin Concentrations Across Pregnancy and Maternal and Child Health: A Systematic Review and Meta-Analysis, Annals of the New York Academy of Sciences. (2019) 1450, no. 1, 47–68, 10.1111/nyas.14093. 30994929 PMC6767572

[39] Gonzales G. F. , Steenland K. , and Tapia V. , Maternal Hemoglobin Level and Fetal Outcome at Low and High Altitudes, American Journal of Physiology-Regulatory, Integrative and Comparative Physiology. (2009) 297, no. 5, R1477–R1485, 10.1152/ajpregu.00275.2009. 19741055 PMC2777782

[40] Smith J. S. , Bullens L. M. , van der Hout-van der Jagt M. B. , van Runnard Heimel P. J. , and Oei S. G. , Effect of Intrapartum Maternal Hemoglobin on Mode of Delivery and Short-Term Neonatal Outcome: A Systematic Review, Obstetrical and Gynecological Survey. (2022) 77, no. 10, 595–605, 10.1097/OGX.0000000000001074. 36242529 PMC9561235

[41] Yao Y. , Sun L. , Zuo X. , Yang Z. , Yang Y. , and Luo J. , A Comparative Study of Different Standards of Iron Supplementation in Pregnant Women with Iron Deficiency in Plateau Area: A Retrospective Cohort Study from a Single Center in China, Medicine (Baltimore). (2025) 104, no. 49, 10.1097/MD.0000000000046327. PMC12688737 41366950

[42] Campbell A. I. K. , Xu Y. , Skeith L. , and Federspiel J. J. , Racial and Ethnic Disparities in Eligibility for Postpartum Venous Thromboembolism Prophylaxis in the United States, Journal of Thrombosis and Haemostasis. (2024) 22, no. 2, 545–552, 10.1016/j.jtha.2023.10.004. 37838240 PMC10872622

[43] Chen Y. , Dai Y. , Song J. et al., Establishment of a Risk Assessment Tool for Pregnancy-Associated Venous Thromboembolism and its Clinical Application: Protocol for a Prospective Observational Study in Beijing, BMC Pregnancy and Childbirth. (2019) 19, no. 1, 10.1186/s12884-019-2448-7. PMC6693270 31409379

